# G protein‐coupled estrogen receptor in the rostral ventromedial medulla contributes to the chronification of postoperative pain

**DOI:** 10.1111/cns.13704

**Published:** 2021-07-13

**Authors:** Jia‐Jia Xu, Po Gao, Ying Wu, Su‐Qing Yin, Ling Zhu, Sai‐Hong Xu, Dan Tang, Chi‐Wai Cheung, Ying‐Fu Jiao, Wei‐Feng Yu, Yuan‐Hai Li, Li‐Qun Yang

**Affiliations:** ^1^ Department of Anesthesiology The First Affiliated Hospital of Anhui Medical University Hefei China; ^2^ Department of Anesthesiology Renji Hospital School of Medicine Shanghai Jiaotong University Shanghai China; ^3^ Department of Anesthesiology The University of Hong Kong Hong Kong China

**Keywords:** chronification, G protein‐coupled estrogen receptor, postoperative pain, rostral ventromedial medulla, μ‐type opioid receptor

## Abstract

**Aims:**

Chronification of postoperative pain is a common clinical phenomenon following surgical operation, and it perplexes a great number of patients. Estrogen and its membrane receptor (G protein‐coupled estrogen receptor, GPER) play a crucial role in pain regulation. Here, we explored the role of GPER in the rostral ventromedial medulla (RVM) during chronic postoperative pain and search for the possible mechanism.

**Methods and Results:**

Postoperative pain was induced in mice or rats via a plantar incision surgery. Behavioral tests were conducted to detect both thermal and mechanical pain, showing a small part (16.2%) of mice developed into pain persisting state with consistent low pain threshold on 14 days after incision surgery compared with the pain recovery mice. Immunofluorescent staining assay revealed that the GPER‐positive neurons in the RVM were significantly activated in pain persisting rats. In addition, RT‐PCR and immunoblot analyses showed that the levels of GPER and phosphorylated μ‐type opioid receptor (p‐MOR) in the RVM of pain persisting mice were apparently increased on 14 days after incision surgery. Furthermore, chemogenetic activation of GPER‐positive neurons in the RVM of *Gper‐Cre* mice could reverse the pain threshold of pain recovery mice. Conversely, chemogenetic inhibition of GPER‐positive neurons in the RVM could prevent mice from being in the pain persistent state.

**Conclusion:**

Our findings demonstrated that the GPER in the RVM was responsible for the chronification of postoperative pain and the downstream pathway might be involved in MOR phosphorylation.

## Graphical abstract

Noxious stimulation induced by plantar incision surgery can upregulate GPER expression in RVM and promote the phosphorylation of MOR, leading to the activation of RVM neurons, which ultimately mediates the chronification of postoperative pain. These findings provide a new target for preventing the transition from acute pain to chronic pain after surgery and treatment of chronic postoperative pain.

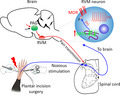

## 1 INTRODUCTION

Postoperative pain is a common puzzle that bothering a large number of perioperative patients.^1^, ^2^ To make matters worse, approximately 10% ‐ 50% patients will suffer the long‐term postoperative pain, which transfers from acute pain and represents a huge potential of chronic pain.^3^, ^4^ This unexplained, persistent, and recurring chronic pain is not sensitive to the existing analgesic measures, which is a hard part of clinical treatment.^4^, ^5^, ^6^ Thus, it is of great clinical significance to study the mechanisms underlying the occurrence and development of postoperative pain and find out a new therapeutic target.

Rostral ventromedial medulla (RVM), as the last functional nucleus of descending pain modulation system in the brain, has been regarded as a gateway that controls the pain regulation signaling transmitted from periaqueductal gray (PAG) to the dorsal horn of the spinal cord, which has a bidirectional role in pain facilitation or inhibition.^7^ It has been proved that the descending modulation of RVM determined whether acute pain will develop into chronic pain to a large extent.^8^, ^9^ Moreover, RVM is also the major site for analgesic effects of endogenous or exogenous opioids,^10^, ^11^, ^12^, ^13^ since silencing the dual GABA/enkephalinergic RVM neurons can substantially increase behavioral sensitivity to heat and mechanical stimuli in adult mice.^14^

G protein‐coupled estrogen receptor (GPER), or recognized as G protein‐coupled receptor 30 (GPR30), has been introduced as the plasma membrane to evoke rapid non‐genomic estrogenic signaling,^15^ which is widely distributed in different organs with the nervous system included.^16^ Some studies have shown that estrogen and its nuclear receptors (ERα and ERβ) play a critical role in pain regulation.^17^, ^18^, ^19^, ^20^ However, few reports have directly investigated estrogenic effects on the descending pain modulation system, especially the function of GPER in the RVM on the chronification of postoperative pain.

Here, we clarified the role of GPER in the RVM on the chronification of postoperative pain and its underlying mechanisms. Initially, we established the model of plantar incision surgery‐induced chronic postoperative pain. Then, in the RVM, the activation of neurons was examined by immunofluorescence staining and the expression of GPER was explored by RT‐PCR and immunoblot analyses. Finally, we investigated the role of GPER‐positive RVM neurons on the chronification of postoperative pain and the phosphorylation of MOR by chemogenetic activation or inhibition of GPER‐positive RVM neurons in *Gper‐Cre* mice.

## 2 MATERIAL AND METHODS

### 2.1 Animals

Adult male C57BL/6J mice, Sprague Dawley rats (Shanghai Jiesjie Laboratory Animal Co., Ltd.; 20–25 g for mice and 180–200 g for rats, 8–10 weeks old) and *Gper‐Cre* transgenic mice (Shanghai Nanfang Model Biotechnology Co., Ltd), were utilized in the experiments. The mice were maintained under a 12‐h light/dark cycle at 22–25℃. All animals were given *ad libitum* access to water and food. The animal experiments were approved by the Ethnic Committee for Experimental Use of Animals of Shanghai Jiaotong University School of Medicine (#SYXK‐2013‐0050) and were conducted in accordance with the Guiding Principles for the Care and Use of Animals in Research, the ARRIVE 2.0 guidelines^21^ and the Animal Management Rule of the Ministry of Public Health, China (documentation 545, 2001).

### 2.2 Plantar incision‐induced pain model

The plantar incision surgery was conducted following the protocol from Pogatzki, E. M, et al.^22^ Under anesthesia with 2% isoflurane, the right hind paw of mouse (or rat) was prepared with iodophor, then a 5 mm (10 mm for rat) longitudinal incision was made through the skin and fascia 2 mm (5 mm for rat) away from the edge of the heel with a sterile No. 11 scalpel. To leave the origin and insert intact, the muscle was elevated using a curved tweezer. The incision was sutured with 8‐0 nylon and then dressed with antibiotic ointment. A homoeothermic blanket was used to maintain the temperature at 37℃ during the surgery.

### 2.3 Behavioral tests

Mechanical and thermal pain thresholds were both tested. Mice were habituated to the test environment for 30 min every day from 3 days before measurement. Mice in quiet state or less autonomous activities were deemed as well acclimatized.

#### 2.3.1 Mechanical allodynia test

Von Frey filaments (0.008–2 g for mouse, 0.5–12 g for rat) were vertically applied to the hind paw adjacent to the incision for 1 s. The same filament was stimulated 5 times with an interval of more than 1 min. Three or more times obvious painful behavioral response such as foot licking or foot withdrawal was considered as effective pain reaction, and the gram was recorded as the mechanical pain threshold.

#### 2.3.2 Hot plate test

Similarly, after the mice were habituated, they were gently placed on the hot plate at 52.5°C (IITC Life Science Inc.). The response latency to either lick, retraction, or jump was recorded as the nociceptive endpoint. In the absence of a response, mice were removed from the hot plate at 30 s to avoid tissue injury. Each mouse was measured 3 times, and the mean value was regarded as the thermal pain threshold.

### 2.4 RVM stereotaxic injection

The *Gper‐Cre* mice were anesthetized with sodium pentobarbital (0.06 mg g^−1^, i.p.) and secured in a stereotactic frame (RWD Instruments). A midline scalp incision was made and a hole was drilled on the skull to allow passage of glass pipette filled with the virus. After drilling the skull of mice, the chemogenetic virus (pAAV‐hSyn‐DIO‐hM3D(Gq)‐mCherry or pAAV‐hSyn‐DIO‐hM4D(Gi)‐mCherry) solution (0.4 μl) was injected into RVM (coordinates: 0 mm from midline, 5.7 mm ventral to skull and 5.88 mm from bregma) at 40 nl min^−1^, allowing an additional 10 min for viral particles to diffuse before the pipette was slowly withdrawn. Finally, the skin of skull surface was sutured with 4‐0 nylon and Iodophor was used to disinfect the wounds.

### 2.5 Drug administration

Clozapine N‐oxide (CNO, 5 mg, Sigma) was initially dissolved with 1 ml saline (0.9%) and stored at 4℃ according to the method previously described.^23^ CNO (3 mg kg^−1^, i.p.) was administrated intraperitoneally per day from 10 to 14 days after incision surgery. An equal volume of 0.9% saline was given to control mice.

### 2.6 Western blot analysis

RVM samples of different groups of mice were extracted and transferred from 10% SDS‐PAGE to nitrocellulose membranes. Following inhibition with 5% skimmed milk for 1 h, they were exposed to different primary antibodies against GPER (1:1000, ABclonal), MOR (1:1000, Novus Biologicals), p‐MOR (1:1000, Cell Signaling Technology), and GAPDH (1:20000, Proteintech) at 4℃ overnight. After combined with secondary antibodies, visualization of immunoreactive bands was performed using an enhanced chemiluminescent reagent (Thermo). The ImageQuant LAS 4000 mini (GE Healthcare Life Sciences) was used to record digital images. After normalization against the loading control GAPDH, the density of each protein band was quantified by Image J software (NIH).

### 2.7 RT‐PCR

The total RNA of RVM were extracted by Vazyme kit and transcribed to cDNA with superscript II and random hexamer primers. PCR amplification was done using the following primers: *Gper* (F: 5′‐CCTCTGCTACTCCCTCATCG‐3′, R: 5′‐ACTATGTGGCCTGTCAAGGG‐3′), *Mor* (F: 5′‐CCAGGGAACATCAGCGACTG‐3′, R: 5′‐CATGGGTCGGACTGGTTGC‐3′), *Gapdh* (F: 5′‐AAGAAGGTGGTGAAGCAGGCATC‐3′, R: 5′‐CGGCATCGAAGGTGGAAGATG‐3′).

### 2.8 Immunofluorescence staining

The protocol of immunofluorescence staining referred to the methods previously described.^24^ After perfused with saline (0.9%) and paraformaldehyde (4%) sequentially, the brainstem and lumbar spinal cord were removed, post‐fixed overnight at 4℃ in paraformaldehyde (4%), and then cryopreserved with sucrose (30%). The coronal sections of brainstem with lumbar spinal cord and RVM were cut at 20 μm, respectively. After blocking for 1 h, each tissue section was successively combined with primary antibody (GPER, 1:1000, LSBio; c‐Fos, 1:1000, Abcam; NeuN, 1:1000, Abcam; GFAP, 1:1000, Cell Signaling Technology; Iba1, 1:500, Abcam) and secondary antibody. Finally, the fluorescence images were recorded with a laser‐scanning confocal microscope (FV3000, Olympus Corp.). C‐Fos staining was chosen to reflect the activated neurons. All images were analyzed by Image J to calculate the number of c‐Fos positive neurons. Data were obtained from three or four animals in each group, and eight slices or ten slices were chosen from each animal (eight slices for RVM, ten slices for spinal cord) to sum up in total.

### 2.9 Statistical analysis

Statistical tests were performed by GraphPad Prism 7.0 (GraphPad Software Inc.). All data are shown as mean ± SEM. Data normality was verified by Shapiro‐Wilk test. The results of behavioral tests were analyzed with two‐way repeated measures ANOVA followed by the Bonferroni post hoc test. One‐way ANOVA analysis followed by the Tukey post hoc test was employed for multiple group comparisons. The level of statistical significance was expressed as *p*‐value <0.05.

## 3 RESULTS

### 3.1 Chronification of acute pain induced by plantar incision surgery

**FIGURE 1:**
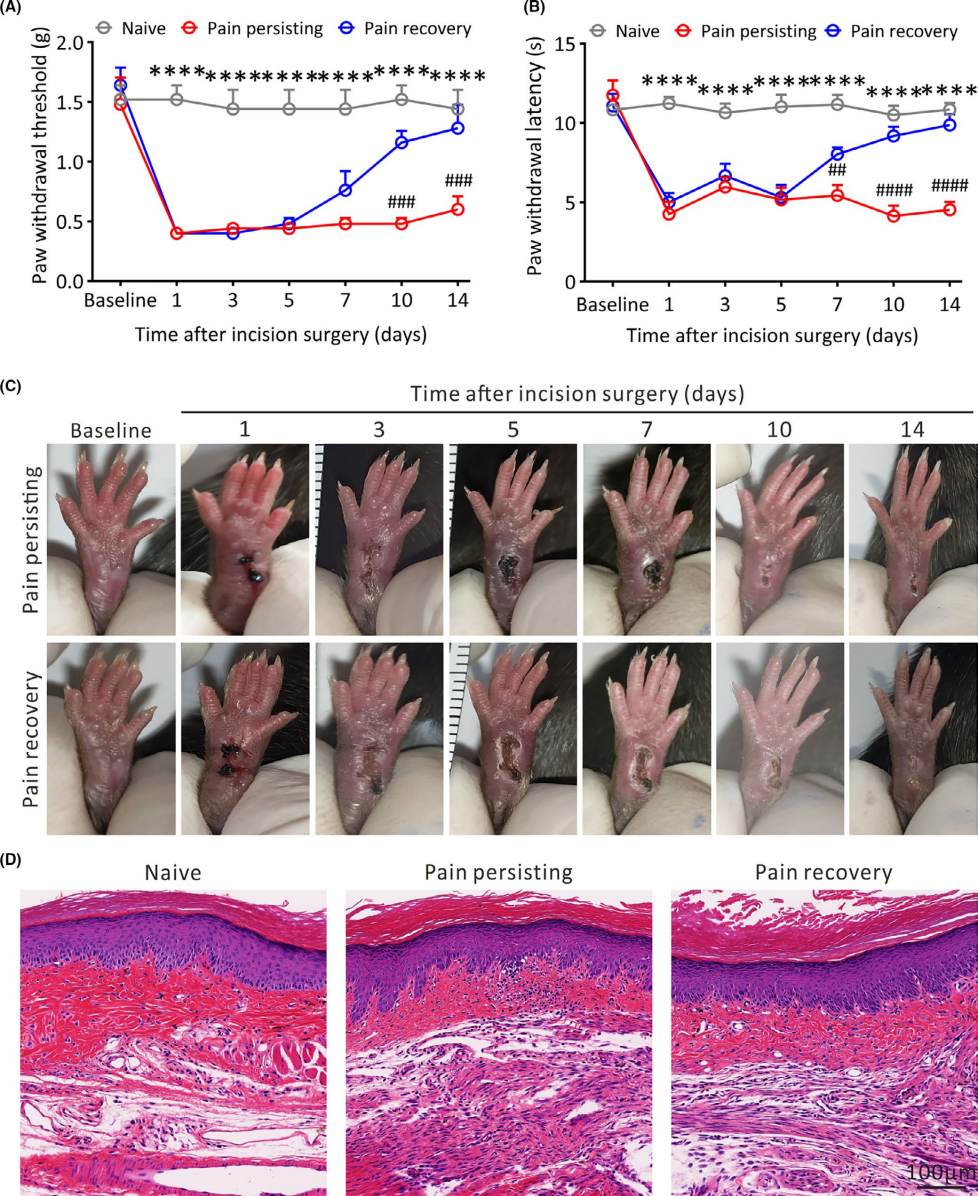
Characters of pain persisting mice and pain recovery mice induced by plantar incision surgery. (A) and (B) The PWT and PWL of mice are tested at baseline, 1, 3, 5, 7, 10 and 14 days after incision surgery (*n* = 5 in each group, ****p < 0.0001, between Naive mice and Pain persisting mice; ##*p* < 0.01, ###*p* < 0.001, ####*p* < 0.0001, between pain persisting mice and pain recovery mice, two‐way repeated measures ANOVA followed by the Bonferroni post hoc test). (C) Photographs of plantar incision of pain persisting and pain recovery mice at different time points. (D) Representative HE staining images of the skin around the plantar incision in different pain states mice

Among perioperative patients with postoperative pain caused by surgeries, a certain proportion will develop into chronic pain.^25^ To better study the mechanisms of chronification of acute postoperative pain, plantar incision was applied to simulate surgical trauma. The behavioral tests with von Frey and hot plate were conducted to detect both thermal and mechanical pain thresholds before the incision surgery (baseline) and 1, 3, 5, 7, 10, 14 days after the incision surgery. Behavioral results showed that there were no obvious changes both in von Frey test (*p* > 0.05, Figure 1A) and hot plate (*p* > 0.05, Figure 1B) between baseline and other time points in naive mice. Compared with naive mice, 1 day after incision surgery, the pain threshold of incision surgery mice was significantly decreased regardless of the mechanical or thermal painful stimulus (*p* < 0.0001) with no difference at the baseline (Figure 1A,B). On day 3–5 after incision surgery, the persistent downtrend of incision mice in withdrawal threshold and latency showed that nociceptive allodynia developed after plantar incision surgery. However, same as the clinical phenomenon, there were two trends of pain development in mice received the same plantar incision: majority (83.8%) of mice began to go up at day 7 and approximately restored to the normal level on day 14 after incision surgery, finally resulted in no significance compared with naive mice, which were recognized as pain recovery group. The other small part (16.2%) developed into pain persisting group with consistent low paw withdrawal latency (PWL) and paw withdrawal threshold (PWT) even 14 days after incision surgery compared with the pain recovery mice, indicating a state of persistent hyperalgesia. There was no obvious difference in the PWT of contralateral paw without incision surgery among three groups of mice (*p* > 0.05, Figure S1). The recovery progress of plantar incision (Figure 1C) was relatively similar between the pain persisting mice and pain recovery mice at different time points. The plantar appearance of naive mice without incision surgery showed almost no change at different time points (*p* > 0.05, Figure S2). Furthermore, HE examinations (Figure 1D) showed no obvious increase of fibrosis and inflammation in the pain persisting mice on 14 days after incision surgery compared with naive mice and pain recovery mice. In this way, we established a model of chronification of acute postoperative pain induced by plantar incision surgery.

### 3.2 The expression of GPER in RVM is significantly increased in the pain persisting mice

**FIGURE 2:**
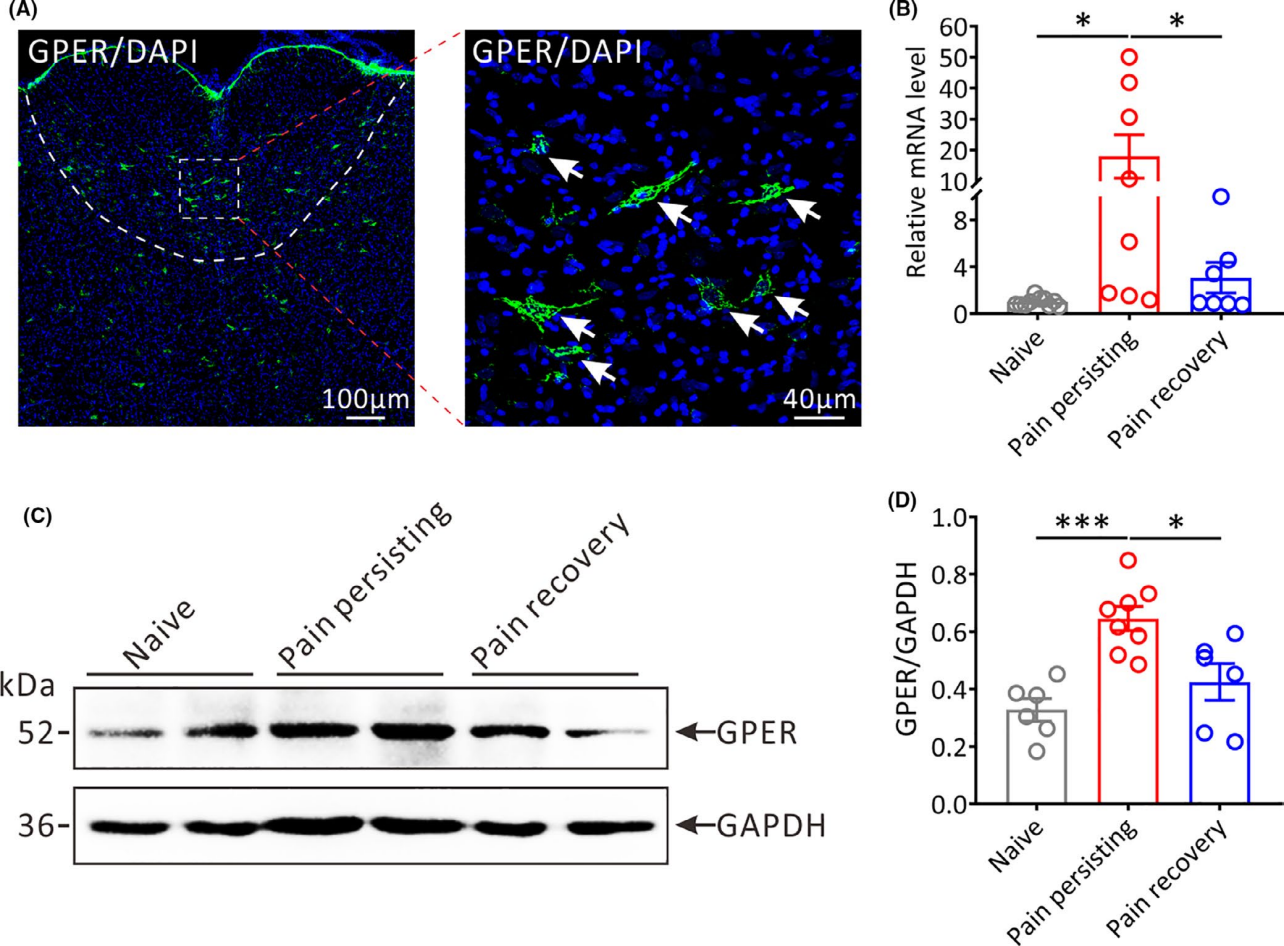
The expression of GPER in the RVM of different pain states mice. (A) Representative immunofluorescence images showing the distribution of GPER‐positive (GPER+) neurons in the RVM. The white arrows show the GPER+ neurons; Green: GPER+ neurons. Scale bars, 100 μm (left) and 40 μm (right). (B) The relative mRNA expression of Gper in the RVM of different pain states mice (Naive: *n* = 10, Pain persisting: *n* = 8, Pain recovery: *n* = 7; *p < 0.05, one‐way ANOVA). (C) Representative Western blot bands showing the expression of GPER in the RVM of different pain states mice (MW: GPER, 52 kDa; GAPDH, 36 kDa). (D) Statistical analysis of the expression of GPER in the RVM of different pain states mice (Naive: *n* = 6, Pain persisting: *n* = 8, Pain recovery: *n* = 6; *p < 0.05, ***p < 0.001, one‐way ANOVA)

Previous research has shown that GPER is abundantly distributed in the central nervous system (CNS) and participates in pain modulation.^26^, ^27^ To best of our knowledge, RVM serves as one of the important functional nuclei for the descending pain modulation in the CNS and is associated with the occurrence and progression of chronic pain.^28^, ^29^ Here, we clarified the distribution of GPER in the RVM by immunofluorescence staining (Figure 2A). Furthermore, compared with naive group mice or pain recovery group mice, the mRNA (Pain persisting vs. Naive, *p* < 0.05; Pain persisting vs. Pain recovery, *p* < 0.05; Figure 2B) and protein (Pain persisting vs. Naive, *p* < 0.001; Pain persisting vs. Pain recovery, *p* < 0.05; Figure 2C,D) levels of GPER in the RVM of pain persisting group mice were apparently increased on 14 days after incision surgery, suggesting that GPER in the RVM can regulate postoperative pain.

### 3.3 The GPER‐positive neurons in RVM are activated in pain persisting rats

**FIGURE 3:**
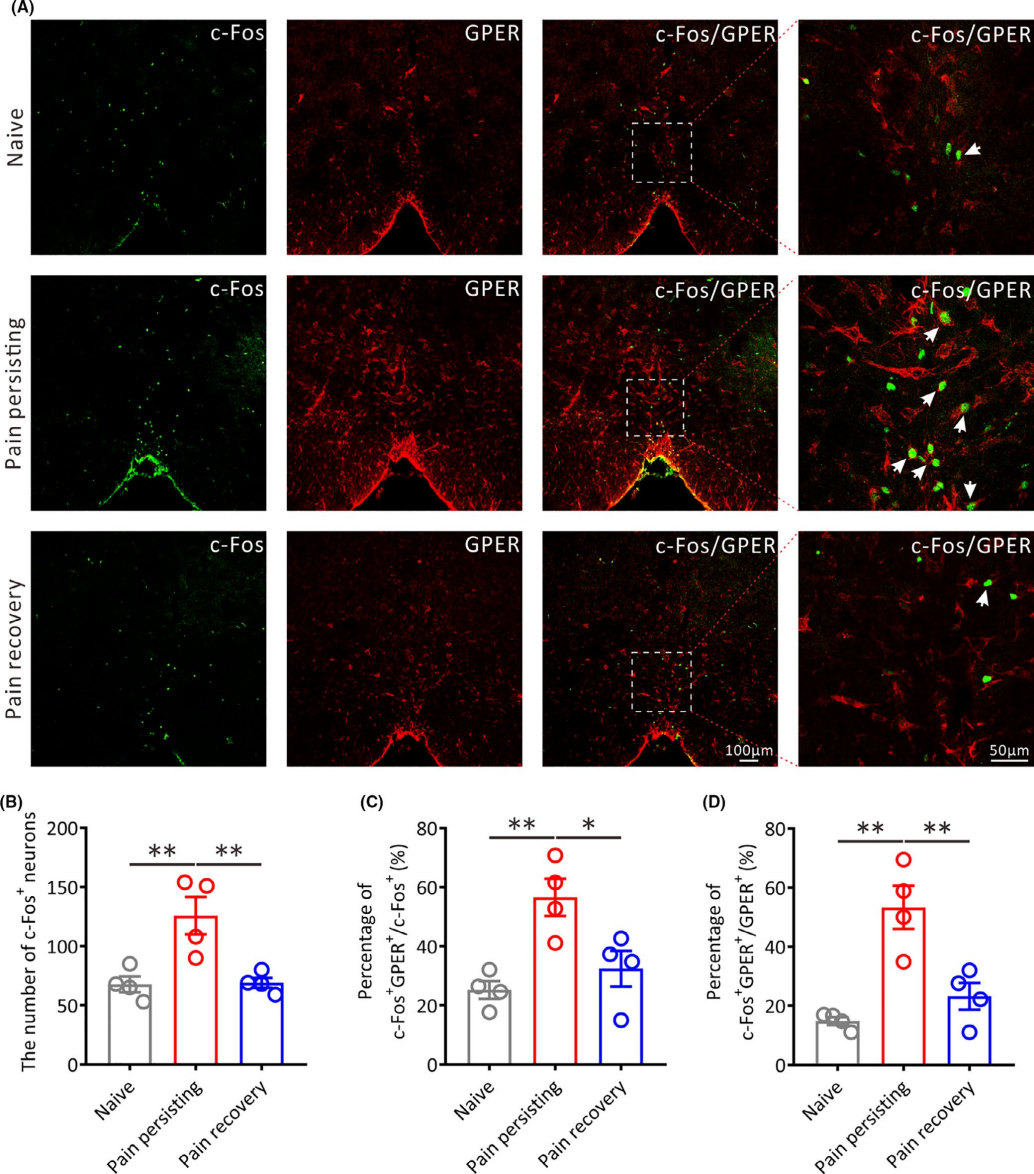
The number of activated GPER‐positive neurons in the RVM under different pain states. (A) Representative immunofluorescence images showing the distribution of c‐Fos+ and GPER+ neurons in the RVM of different pain states on 14 days after incision surgery. The white arrows show the c‐Fos/GPER double positive (c‐Fos+/GPER+) neurons. Green: c‐Fos+ neurons. Red: GPER+ neurons. All scale bars, 100 μm or 50 μm. (B) Statistical analysis of the number of c‐Fos+ neurons in the RVM of different pain states rats (*n* = 4 in each group, **p < 0.01, one‐way ANOVA). (C) Statistical analysis of the percentage of c‐Fos+/GPER+ neurons in total c‐Fos+ neurons in the RVM of different pain states rats (*n* = 4 in each group, **p < 0.01, *p < 0.05, one‐way ANOVA). (D) Statistical analysis of the percentage of c‐Fos+/GPER+ neurons in total GPER+ neurons in the RVM of different pain states rats (*n* = 4 in each group, **p < 0.01, one‐way ANOVA)

We then explored the activation of GPER neurons in the RVM under different pain states by c‐Fos/GPER double immunofluorescence staining (Figure 3A). Our results demonstrated that the amount of c‐Fos‐positive (c‐Fos^+^) neurons in the RVM of pain persisting rats was remarkably higher compared with naive rats or pain recovery rats, respectively (Pain persisting vs. Naive, *p* < 0.01; Pain persisting vs. Pain recovery, *p* < 0.01; Figure 3B). Moreover, the percentage of c‐Fos/GPER double‐positive (c‐Fos^+^/NeuN^+^) neurons of pain persisting rats in c‐Fos^+^ neurons (Pain persisting vs. Naive, *p* < 0.01; Pain persisting vs. Pain recovery, *p* < 0.05; Figure 3C) or GPER^+^ neurons (Pain persisting vs. Naive, *p* < 0.01; Pain persisting vs. Pain recovery, *p* < 0.01; Figure 3D) was markedly higher compared with naive rats or pain recovery rats, respectively, suggesting that the activated GPER neurons in RVM can play a vital role in the chronification of postoperative pain. Furthermore, the type of activated cells in RVM are neurons rather than neuroglial cells, such as astrocytes and microglias (Figure S3).

### 3.4 Phosphorylation of MOR in the RVM is increased in the pain persisting mice

**FIGURE 4:**
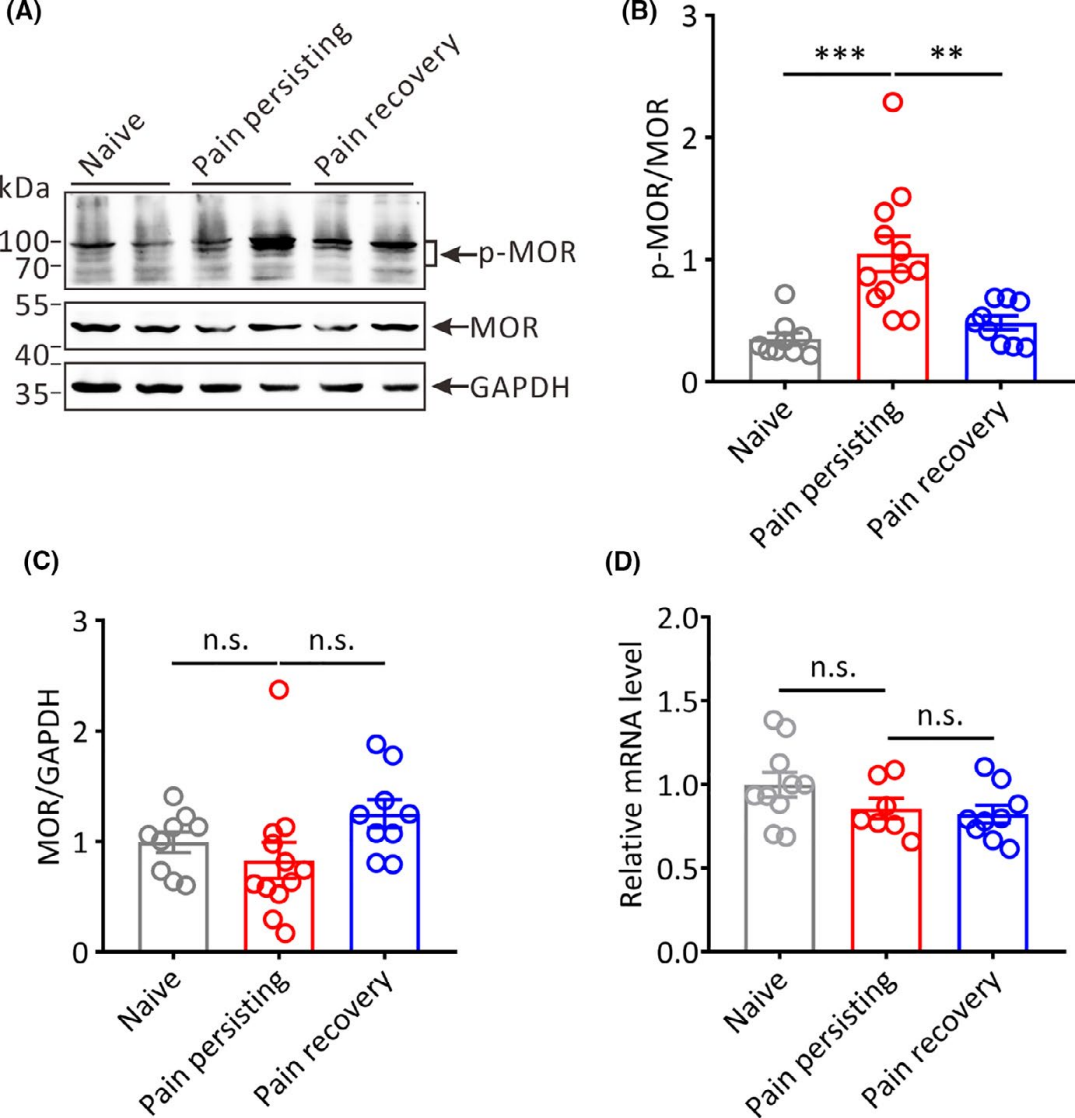
The expression of p‐MOR and MOR in the RVM of different pain states mice. (A) Representative Western blot bands showing the expression of p‐MOR and MOR in the RVM of different groups mice (MW: p‐ MOR, 70–100 kDa; MOR, 45 kDa; GAPDH: 36 kDa). (B) and (C) Statistical analysis of the expression of p‐ MOR and MOR in the RVM in different pain states mice (Naive: *n* = 9, Pain persisting: *n* = 12, Pain recovery: *n* = 9; **p < 0.01, ***p < 0.001, n.s.: no statistical difference, one‐way ANOVA). (D) The relative mRNA expression of Mor in the RVM of different pain states mice (Naive: *n* = 10, Pain persisting: *n* = 7, Pain recovery: *n* = 9; n.s.: no statistical difference, one‐way ANOVA)

Among the pain‐related nuclei in the CNS, RVM is a main area where endogenous and exogenous opioids exert analgesic functions^13^ with high expression of MOR.^30^, ^31^ Previous study has reported that there is a subset of neurons in RVM‐expressed MOR for maintaining thermal hyperalgesia in neuropathic pain rats.^32^ In addition, activation of MOR in RVM can produce antinociception.^33^ To further assess the effect of MOR on the chronification of postoperative pain, the phosphorylation of MOR in the RVM was detected by immunoblotting (Figure 4A). The results demonstrated that the ratio of p‐MOR to MOR was increased significantly in the pain persisting mice compared with naive mice or pain recovery mice (Pain persisting vs. Naive, *p* < 0.001; Pain persisting vs. Pain recovery, *p* < 0.01; Figure 4B), although there was no difference among three groups of mice on the expression of MOR (Pain persisting vs. Naive, *p* > 0.05; Pain persisting vs. Pain recovery, *p* > 0.05; Figure 4C). Similarly, the mRNA level of *Mor* showed no significant difference in different pain states mice (Pain persisting vs. Naive, *p* >.05; Pain persisting vs. Pain recovery, *p* > 0.05; Figure 4D). Collectively, these results indicate that the increased level of p‐MOR in RVM is associated with the chronification of postoperative pain.

### 3.5 Chemogenetic activation or inhibition of GPER‐positive neurons in the RVM affects the chronification of postoperative pain

**FIGURE 5:**
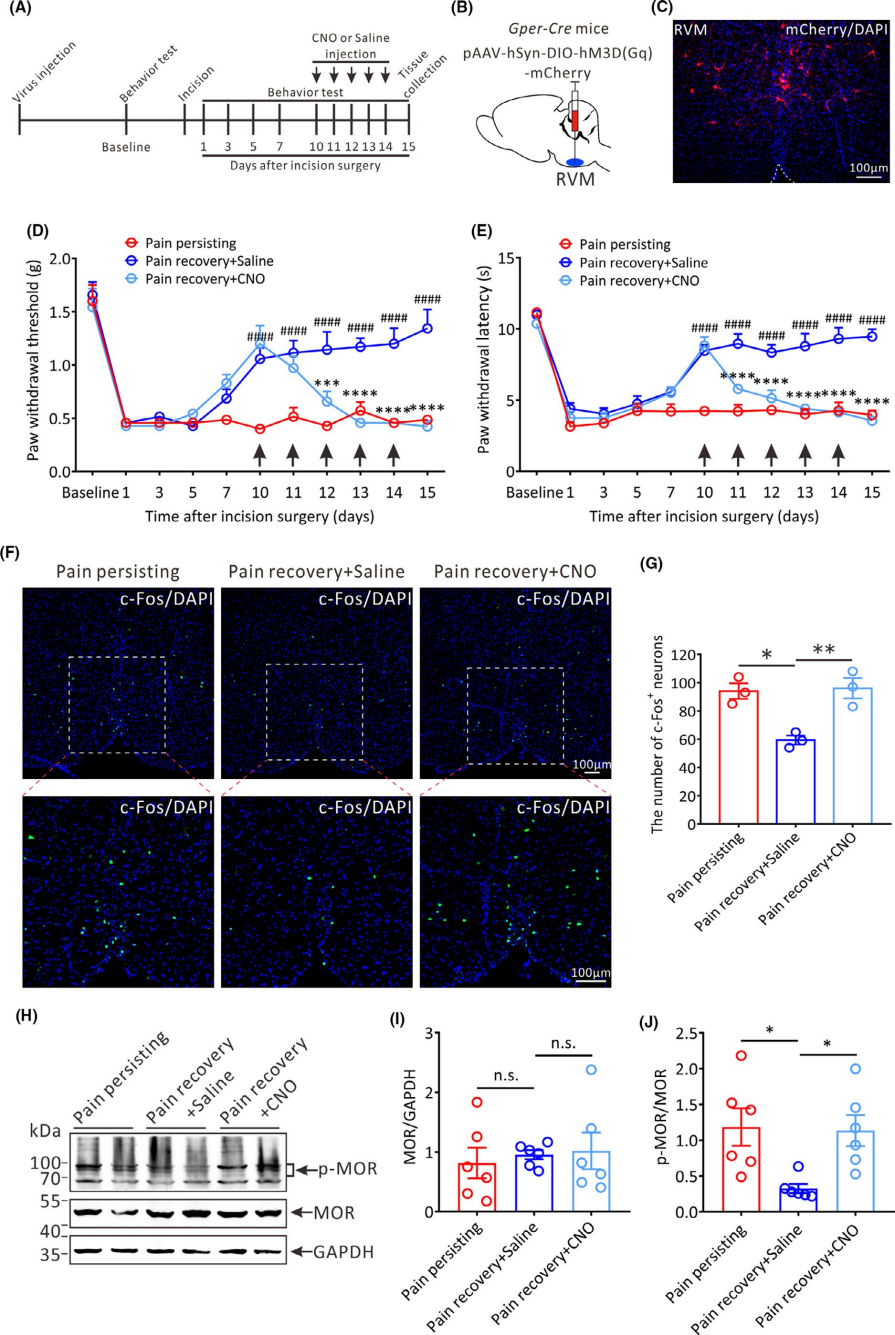
The effects of chemogenetic activation of RVM GPER‐positive neurons on the pain threshold and MOR phosphorylation in the RVM of pain recovery mice. (A) Protocol of the experiments. CNO (3 mg kg^−1^ per day, i.p.) or saline (i.p.) injection is performed from 10 to 14 days after incision surgery. (B) The schematic showing the injection of pAAV‐hSyn‐DIO‐hM3D(Gq)‐mCherry into the RVM of Gper‐Cre mice. (C) Successful expression of hM3D(Gq)‐mCherry in the RVM after viral infection. Scale bar, 100 μm. (D) and (E) The PWT and PWL of the daily baseline before CNO or saline administration in three groups mice: Pain persisting mice, Pain recovery + Saline mice and Pain recovery + CNO mice (*n* = 7 in each group, ***p < 0.001, ****p < 0.0001, between Pain recovery + CNO mice and Pain recovery + Saline mice; ####*p* < 0.0001, between Pain recovery + Saline mice and Pain persisting mice, two‐way repeated measures ANOVA followed by the Bonferroni post hoc test). (F) Representative immunofluorescence images showing the distribution of c‐Fos+ neurons in the RVM of the three groups mice on 15 days after incision surgery. Green: c‐Fos +neurons. All scale bars = 100 μm. (G) Statistical analysis of the number of c‐Fos+ neurons in the RVM of three groups mice (*n* = 3 in each group, *p < 0.05, **p < 0.01, one‐way ANOVA). (H) Representative Western blot bands showing the expression of p‐MOR and MOR in the RVM of three groups mice. (I) and (J) Statistical analysis of the expression of MOR and p‐MOR in the RVM in three groups mice (*n* = 6 in each group, *p < 0.05, n.s.: no statistical difference, one‐way ANOVA)

To further verify the role of GPER in the RVM during pain chronification, we used chemogenetic method to specifically activate GPER‐positive RVM neurons of *Gper‐Cre* mice. *Gper‐Cre* mice were constructed by CRISPR/Cas9 technique and identified by PCR combined with agarose gel electrophoresis (Figure S4). The schematic diagram for animal preparation and behavioral tests were illustrated in Figure 5A. *Gper‐Cre* mice were microinjected with pAAV‐hSyn‐DIO‐hM3D(Gq)‐mCherry into the RVM (Figure 5B), and the successful transfection of viruses in the RVM was showed in the Figure 5C. Plantar incision surgery was performed 3 weeks after viral infection.

With daily intraperitoneal administration of CNO or saline into pain recovery mice from 10 to 14 days after incision surgery, behavioral tests were conducted at 30 min after CNO/saline administration and on the next day before CNO/saline administration again, respectively. We observed that both PWT and PWL showed a significant decline 30 min after CNO administration on 10 and 11 days after incision surgery, whereas both PWT and PWL showed almost no changes after saline administration (Figure S5A,B). Furthermore, the significant difference between before CNO administration and 30 min after CNO administration disappeared from 12 days after incision surgery because the daily baseline of pain thresholds has decreased in CNO‐treated mice before CNO administration (Figure S5A,B). More importantly, we found that the daily mechanical and thermal pain thresholds tested before CNO or saline administration were both gradually decreased in CNO‐treated pain recovery mice from 11 to 15 days after incision surgery compared with the saline‐treated pain recovery mice (Figure 5D,E), suggesting that sustained activation of GPER‐positive neurons in the RVM by repetitive CNO administration can reverse pain threshold of pain recovery mice and restore the phenotype of chronic postoperative pain. Consistent with behavioral results, the amount of c‐Fos‐positive RVM neurons in CNO‐treated mice on 15 days after incision surgery was significantly increased compared with saline‐treated mice (Pain persisting vs. Pain recovery + Saline, *p* < 0.05; Pain recovery + CNO vs. Pain recovery + Saline, *p* < 0.01; Figure 5F,G), suggesting that RVM neurons are activated.

To investigate the effects of activation of GPER‐positive neurons on the phosphorylation of MOR in the RVM, the expression of p‐MOR and MOR was detected by Western blot (Figure 5H). The expression of MOR showed no significant difference in the three groups of mice (Pain persisting vs. Pain recovery + Saline, *p* > 0.05; Pain recovery + CNO vs. Pain recovery + Saline, *p* > 0.05; Figure 5I), whereas the ratio of p‐MOR to MOR in CNO‐treated mice and pain persisting mice were both significantly increased compared with saline‐treated mice on 15 days after incision surgery (Pain persisting vs. Pain recovery + Saline, *p* < 0.05; Pain recovery + CNO vs. Pain recovery + Saline, *p* < 0.05; Figure 5J). These results imply that GPER‐positive neurons in the RVM can facilitate the phosphorylation of MOR, which is an underlying mechanism for the recurrence of postoperative pain in pain recovery mice.

**FIGURE 6:**
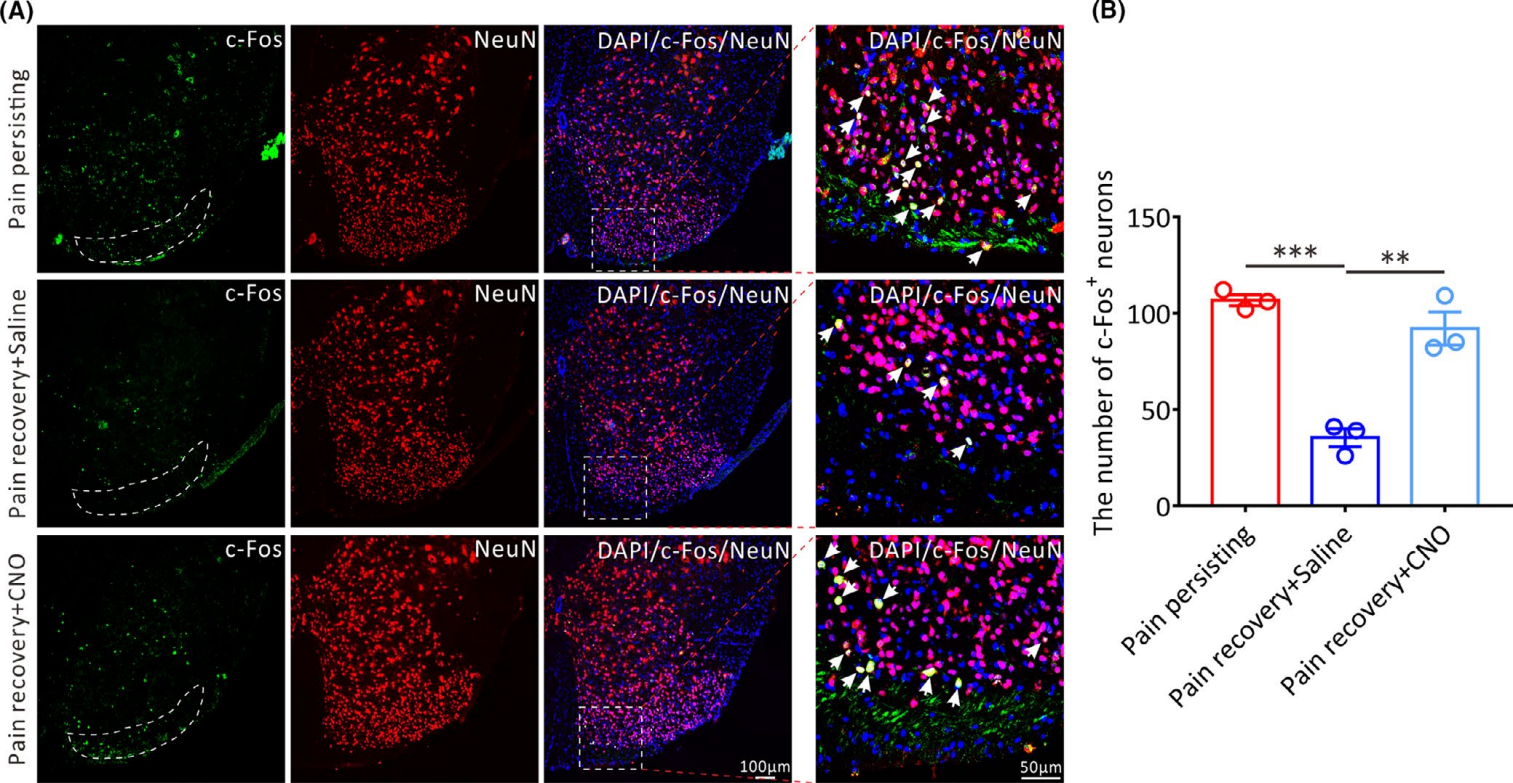
The effect of chemogenetic activation of RVM GPER‐positive neurons on the activation of lumbar spinal dorsal horn neurons in pain recovery mice. (A) Representative immunofluorescence images showing the distribution of c‐Fos+ neurons in the spinal dorsal horn of the three groups mice on 15 days after incision surgery. The white arrows show the c‐Fos+/NeuN+ neurons. Green: c‐Fos+ neurons. Red: NeuN+ neurons. All scale bar, 100 μm or 50 μm. (B) Statistical analysis of the number of c‐Fos+ neurons in the lumbar spinal dorsal horn of three groups mice (*n* = 3 in each group, each mouse was chosen 10 slices, **p < 0.01, ***p < 0.001, one‐way ANOVA)

The rostral ventromedial medulla (RVM) has been regarded as an essential brainstem structure that exhibits descending pain modulation via projections to the spinal dorsal horn. Therefore, we explored the effects of chemogenetic activation of GPER‐positive neurons on the activation of dorsal horn neurons. The results showed that the amounts of c‐Fos^+^ neurons in the dorsal horn of lumbar spinal cord in pain persisting and pain recovery mice treated by CNO were both higher than that in saline‐treated mice (Pain persisting vs. Pain recovery + Saline, *p* < 0.001; Pain recovery + CNO vs. Pain recovery + Saline, *p* < 0.01; Figure 6A,B).

Additionally, we also explored the effects of chemogenetic activation of RVM GPER‐positive neurons on pain persisting mice. We found that both PWT and PWL showed a significant decline 30 min after CNO administration only on the first day of CNO administration and subsequently passed off (Figure S6A,B). Furthermore, the behavioral results showed that the daily PWT and PWL tested before CNO or saline administration were both gradually decreased in CNO‐treated mice from 13 to 15 days after incision surgery and showed a significant difference on 15 days compared with the saline‐treated mice (PWT: Pain persisting + Saline vs. Pain persisting + CNO, *p* < 0.05; PWL: Pain persisting + Saline vs. Pain persisting + CNO, *p* < 0.05; Figure S6C,D), suggesting that sustained activation of RVM GPER‐positive neurons can aggravate the pain behavior of pain persisting mice.

**FIGURE 7:**
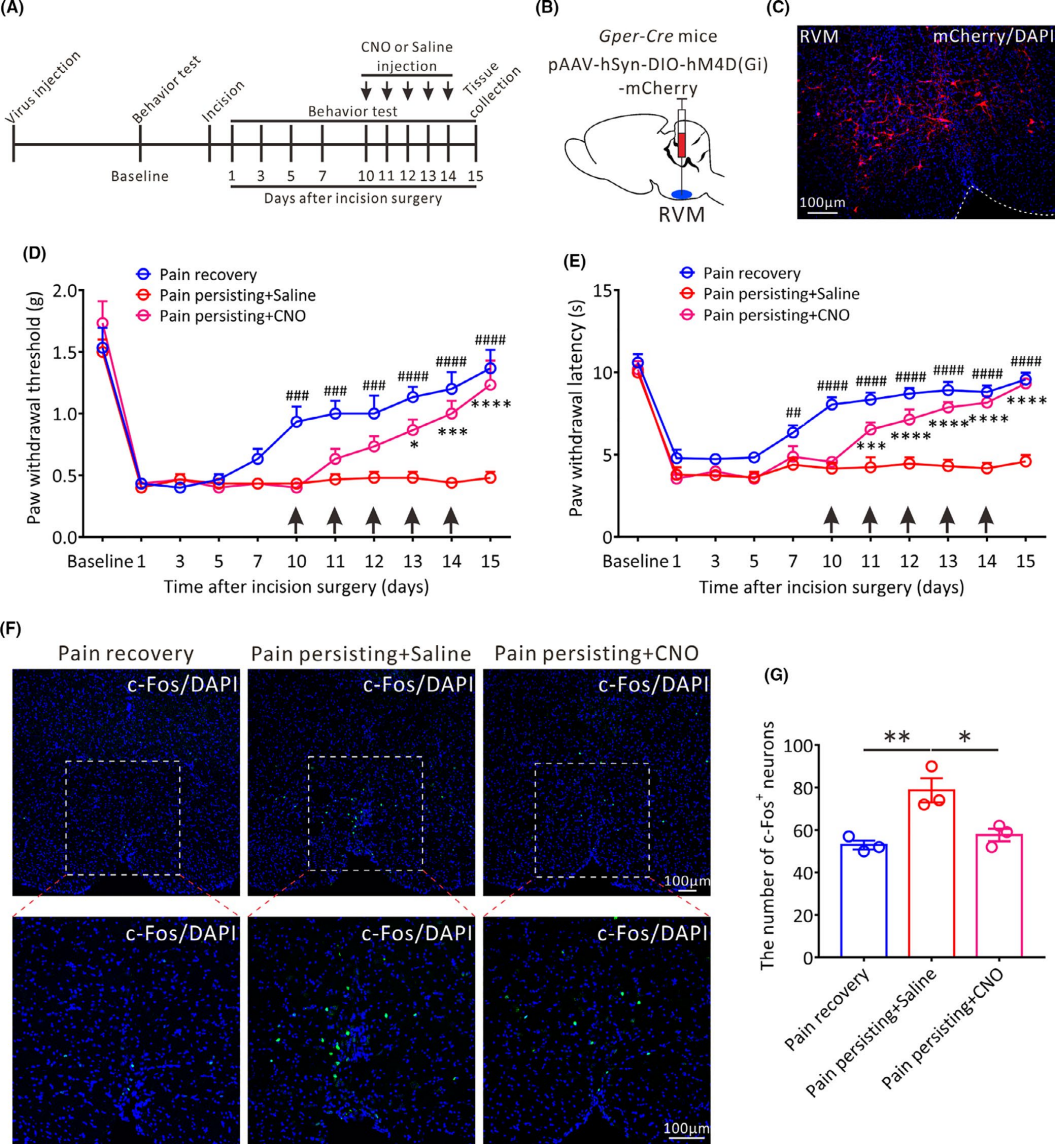
The effects of chemogenetic inhibition of GPER‐positive neurons in the RVM on the pain threshold and the activity of RVM neurons in pain persisting mice. (A) The protocol of the experiments. CNO (3 mg·kg‐ 1 per day, i.p.) or saline (i.p.) injection is performed from 10 to 14 days after incision surgery. (B) The schematic showing the injection of pAAV‐hSyn‐DIO‐hM4D(Gi)‐mCherry into the RVM of Gper‐Cre mice. (C) Successful expression of hM4D(Gi)‐mCherry in the RVM after viral infection. Scale bar, 100 μm. (D) and (E) The PWT and PWL of the daily baseline before CNO or saline administration in three groups mice: Pain recovery mice, Pain persisting + Saline mice and Pain persisting + CNO mice (*n* = 6 in each group, *p < 0.05, ***p < 0.001, ****p < 0.0001, between Pain persisting + CNO mice and Pain persisting + Saline mice; ##*p* < 0.01, ###*p* < 0.001, ####*p* < 0.0001, between Pain recovery mice and Pain persisting + Saline mice, two‐way repeated measures ANOVA followed by the Bonferroni post hoc test). (F) Representative immunofluorescence images showing the distribution of c‐Fos+ neurons in the RVM of the three groups mice on 15 days after incision surgery. Green: c‐Fos+ neurons. All scale bars = 100 μm. (G) Statistical analysis of the number of c‐Fos+ neurons in the RVM of three groups mice (*n* = 3 in each group, *p < 0.05, **p < 0.01, one‐way ANOVA)

We next studied the effects of chemogenetic inhibition of GPER‐positive neurons in the RVM on chronification of postoperative pain. We selectively inhibited the excitability of GPER‐positive neurons by microinjecting pAAV‐hSyn‐DIO‐hM4D(Gi)‐mCherry into the RVM of *Gper‐Cre* mice (Figure 7A,B). The successful viral infection was showed in Figure 7C. With daily intraperitoneal administration of CNO or saline into pain persisting mice from 10 to 14 days after incision surgery, behavioral tests were conducted at 30 min after CNO/saline administration and on the next day before CNO/saline administration again, respectively. The significant increases in PWT and PWL were observed 30 min after CNO administration only on the first day of CNO administration and subsequently passed off because of the increased baseline (Figure S7). Furthermore, the behavioral results showed that the daily PWT and PWL tested before CNO or saline administration were both gradually climbed up in CNO‐treated mice from 11 to 15 days after incision surgery and showed a significant difference on 15 days after incision surgery compared with the saline‐treated mice (PWT: Pain persisting + CNO vs. Pain persisting + Saline, *p* < 0.0001; PWL: Pain persisting + CNO vs. Pain persisting + Saline, *p* < 0.0001; Figure 7D,E), suggesting that sustained inhibition of GPER‐positive neurons in the RVM by repetitive CNO administration can obviously elevate the pain threshold of pain persisting mice and avoid the occurrence of chronic postoperative pain.

Furthermore, we assessed the effects of chemogenetic inhibition of GPER‐positive neurons on the activities of RVM neurons in pain persisting mice. We found that the amount of c‐Fos‐positive RVM neurons in CNO‐treated mice on 15 days after incision surgery was remarkably decreased compared with saline‐treated mice (Pain persisting + CNO vs. Pain persisting + Saline, *p* < 0.05; Pain persisting + Saline vs. Pain recovery, *p* < 0.01; Figure 7F,G), suggesting that the activity of RVM neurons is inhibited in pain persisting mice. Taken together, these findings demonstrate that the GPER‐positive neurons in RVM indeed play a vital role during the chronification of postoperative pain.

Finally, we investigated the effects of chemogenetic inhibition of RVM GPER‐positive neurons on pain recovery mice. The significant increases in PWT and PWL were observed 30 min after CNO administration only on the first day of CNO administration and subsequently passed off (Figure S8A,B). Moreover, we found that the daily PWT and PWL tested before CNO or saline administration were both gradually climbed up in CNO‐treated mice from 11 to 15 days after incision surgery and showed a significant difference on 15 days compared with the saline‐treated mice (PWT: Pain recovery + Saline vs. Pain recovery + CNO, *p* < 0.05; PWL: Pain recovery + Saline vs. Pain recovery + CNO, *p* < 0.05; Figure S8C,D), suggesting that sustained inhibition of RVM GPER‐positive neurons can significantly accelerate the recovery of pain threshold in pain recovery mice.

## 4 DISCUSSION

Acute pain is almost ubiquitous after surgery, and it can be controlled and alleviated within 1 week. Unfortunately, for some patients, acute postoperative pain persists beyond the normal time of tissue healing and converts into the chronic pain state.^34^, ^35^, ^36^ Chronic postoperative pain is increasingly recognized as a public health problem not only because of the distress, discomfort and cost burden, but also because most of the current approaches used to manage this have contributed substantially to the opioid crisis.^37^ However, the mechanisms of occurrence and progression of chronic postoperative pain in the descending pain modulation system are still unclear, especially in RVM. The current study has provided compelling evidences that RVM neurons are significantly activated in pain persisting animals with significant increase in the expression of GPER. Chemogenetic results show that GPER‐positive neurons in RVM play a crucial role in the chronification of postoperative pain and the underlying mechanism involves the phosphorylation of MOR. The findings are expected to play a guiding role in the prevention and treatment of chronic postoperative pain.

In this study, we used the plantar incision pain model to simulate the acute pain and the transition from acute pain to chronic pain (TAPCP) following common surgeries, such as breast and thoracic surgery, orthopedic surgery, limb surgery, and arterial bypass surgery. Currently, the thorough mechanisms of TAPCP after surgery are complex and poorly understood. Previous research has shown that peripheral and central sensitization are crucial in TAPCP as well as the persistent postoperative pain.^5^, ^38^ In general, continuous or intense peripheral nociceptive stimulation may induce central sensitization. The RVM, composed of the adjacent reticular formation and nucleus raphe magnus, has been regarded as a key player in the descending pain modulation systems.^28^ It receives information from different central nuclei, including PAG, parabrachial complex (PB), and hypothalamus.^8^ The descending PAG‐RVM pathway, which modulates ascending nociceptive transmission at the spinal cord dorsal horn, is involved in the occurrence of chronic pain.^39^ Many evidences suggest that an imbalance between descending facilitation and descending inhibition, particularly heightened descending facilitation from RVM, may underlie central sensitization in various pathological pain conditions.^40^, ^41^, ^42^, ^43^, ^44^, ^45^ Our results showed that the number of activated GPER^+^ neurons of RVM in chronic postoperative pain rats was remarkably higher compared with pain recovery rats or naive rats, suggesting that GPER^+^ neurons in the RVM can regulate the chronification of postoperative pain.

Perioperative patients often trend to feel anxious or depressed generally summarized as the perioperative negative emotions.^46^, ^47^ Accumulating evidence suggests that negative emotions may promote the susceptibility of chronic postoperative pain.^48^ Besides, the secreted estrogen in the CNS is closely related to negative emotions and exerts functions when combined with GPER.^49^, ^50^ In our previous study, the regulation of estrogen synthase aromatase on visceral pain has been investigated,^51^ also indicating to further study the roles of GPER in the occurrence and progression of chronic postoperative pain. Over recent years, there has been a growing attention on the effects of GPER regulating pain. As reported by Luo et al., GPER may accelerate the development of bone cancer pain through suppressing inhibitory transmission and inducing excitatory transmission in the spinal cord.^52^ In addition, GPER in the basolateral amygdala is closely associated with pain‐related anxiety.^49^ Furthermore, GPER antagonist G15 could efficiently suppress formalin‐induced spontaneous biphasic nociceptive responses.^53^ However, the role of GPER in the RVM during pain regulation has not been studied. Based on these, we investigated that the expression of GPER in the RVM under different postoperative pain states and observed that GPER was highly expressed in mice with chronic postoperative pain. Moreover, we demonstrated that GPER‐positive neurons in the RVM could regulate the chronification of postoperative pain by chemogenetic techniques.

Many studies have shown that the dysfunction of the endogenous opioid analgesic system is responsible for chronic pain.^54^, ^55^, ^56^ The activation of MOR by opioids, such as morphine, tends to occur at the RVM where it exerts analgesic effects.^31^, ^57^, ^58^, ^59^ Maione et al. have founded that the interaction between MOR and TRPV1 in the RVM can induce analgesia and activate glutamate transmission.^60^ Furthermore, physical activity can activate MOR in RVM to prevent the increase in paw withdrawal frequency through modulation of serotonin transporter (SERT).^61^ Besides, it has been proved that estrogen may negatively affect MOR‐mediated signaling in the dentate gyrus, the hypothalamus, and the medial preoptic nucleus, probably via increasing phosphorylation of MOR.^62^, ^63^, ^64^, ^65^ Our study revealed that the ratio of p‐MOR to MOR was markedly elevated in the RVM of chronic postoperative pain mice compared with pain recovery mice and that the ratio of p‐MOR to MOR was restored in pain recovery mice by chemogenetic activation of GPER‐positive neurons in the RVM. These results suggest that increased phosphorylation of MOR is a potential mechanism of chronification of postoperative pain. Additionally, many recent studies have indicated that certain cytokines and chemokines as well as inflammation pathways are involved in the molecular regulation of neuropathic pain,^66^, ^67^, ^68^ which provided a speculation that the regulation of GPER in chronic postoperative pain may also involve inflammatory signaling mechanisms. We will explore this speculation in future study.

In conclusion, our findings provided strong evidence that GPER expression and the number of activated GPER^+^ neurons were both upregulated in the RVM of chronic postoperative pain animals. Chemogenetic activation of GPER‐positive neurons makes the pain recovery mice present the phenotype of chronic postoperative pain, and the underlying mechanism may be to promote the phosphorylation of MOR. These findings indicate that GPER can serve as a new target for preventing TAPCP after surgery and treating chronic postoperative pain.

## CONFLICT OF INTEREST

The authors declare no conflict of interest.

## Supporting information

Fig S1

Fig S2

Fig S3

Fig S4

Fig S5

Fig S6

Fig S7

Fig S8

Supplementary Material

## Data Availability

The data that support the findings of this study are available from the corresponding author upon reasonable request.
