# Supplementary figures and images for: G protein‐coupled estrogen receptor in the rostral ventromedial medulla contributes to the chronification of postoperative pain
Source: CNS Neurosci Ther. 2021 Jul 13;27(11):1313–26. doi: 10.1111/cns.13704 (PMC8504531; doi:10.1111/cns.13704)

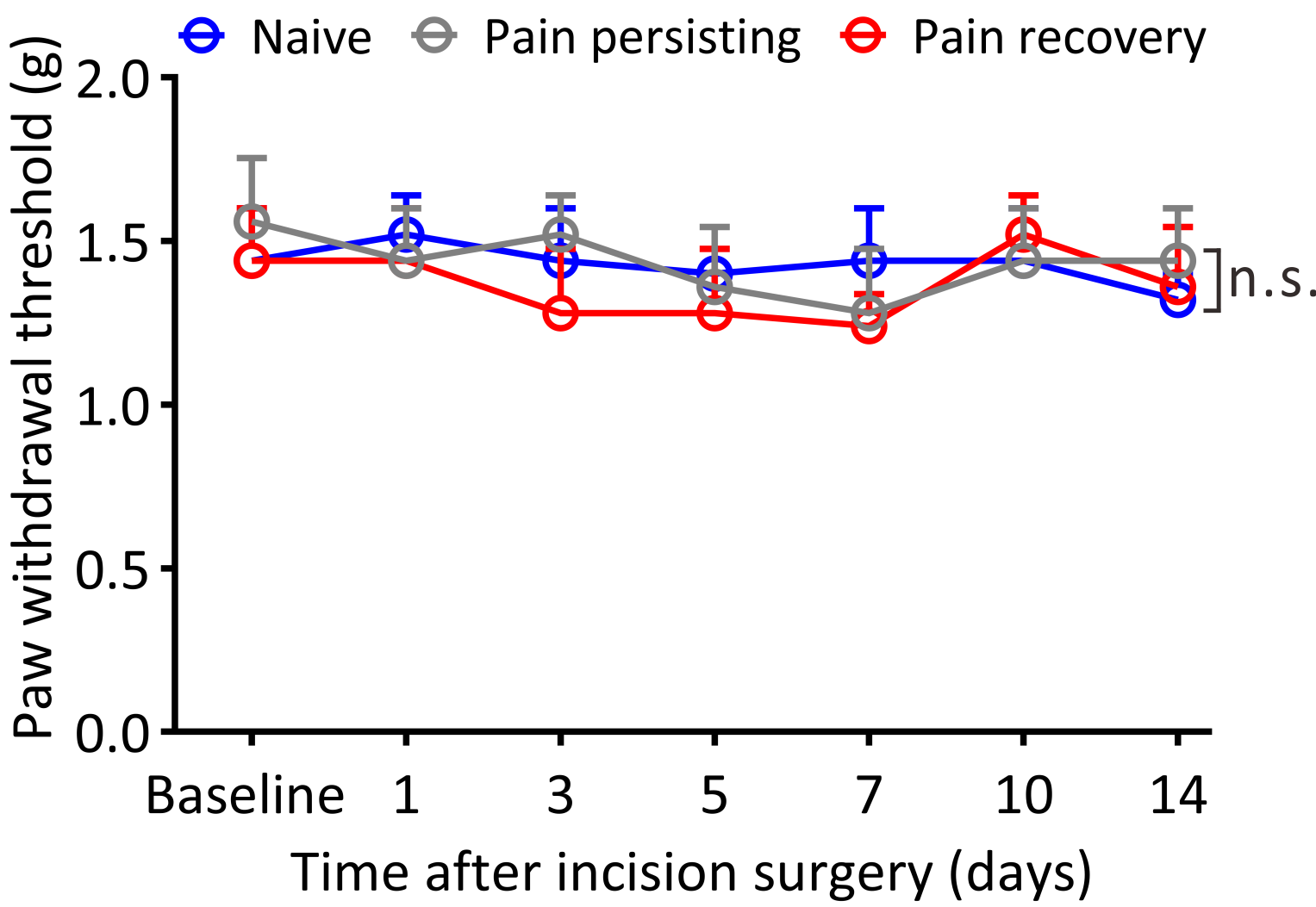

Supplement: Supplementary file 1 — Fig S1 [file CNS-27-1313-s008.tif]

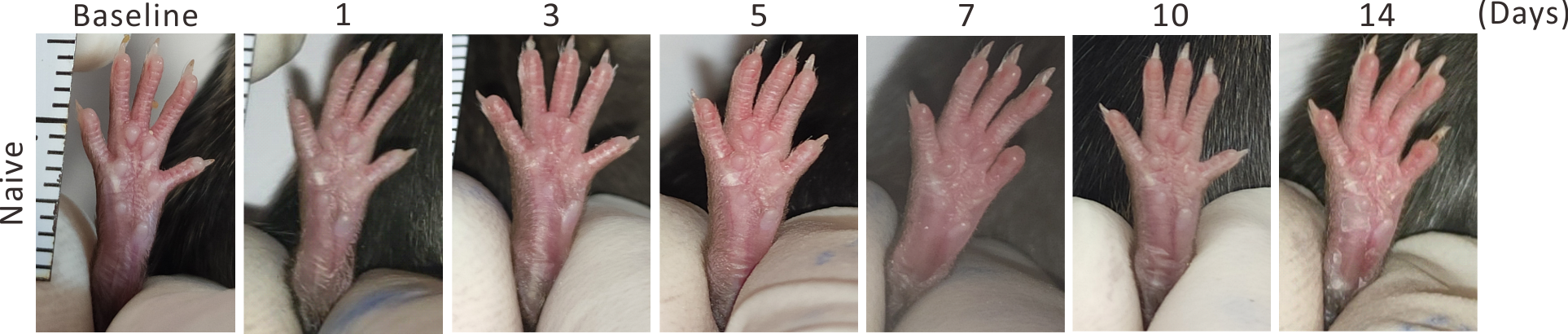

Supplement: Supplementary file 2 — Fig S2 [file CNS-27-1313-s004.tif]

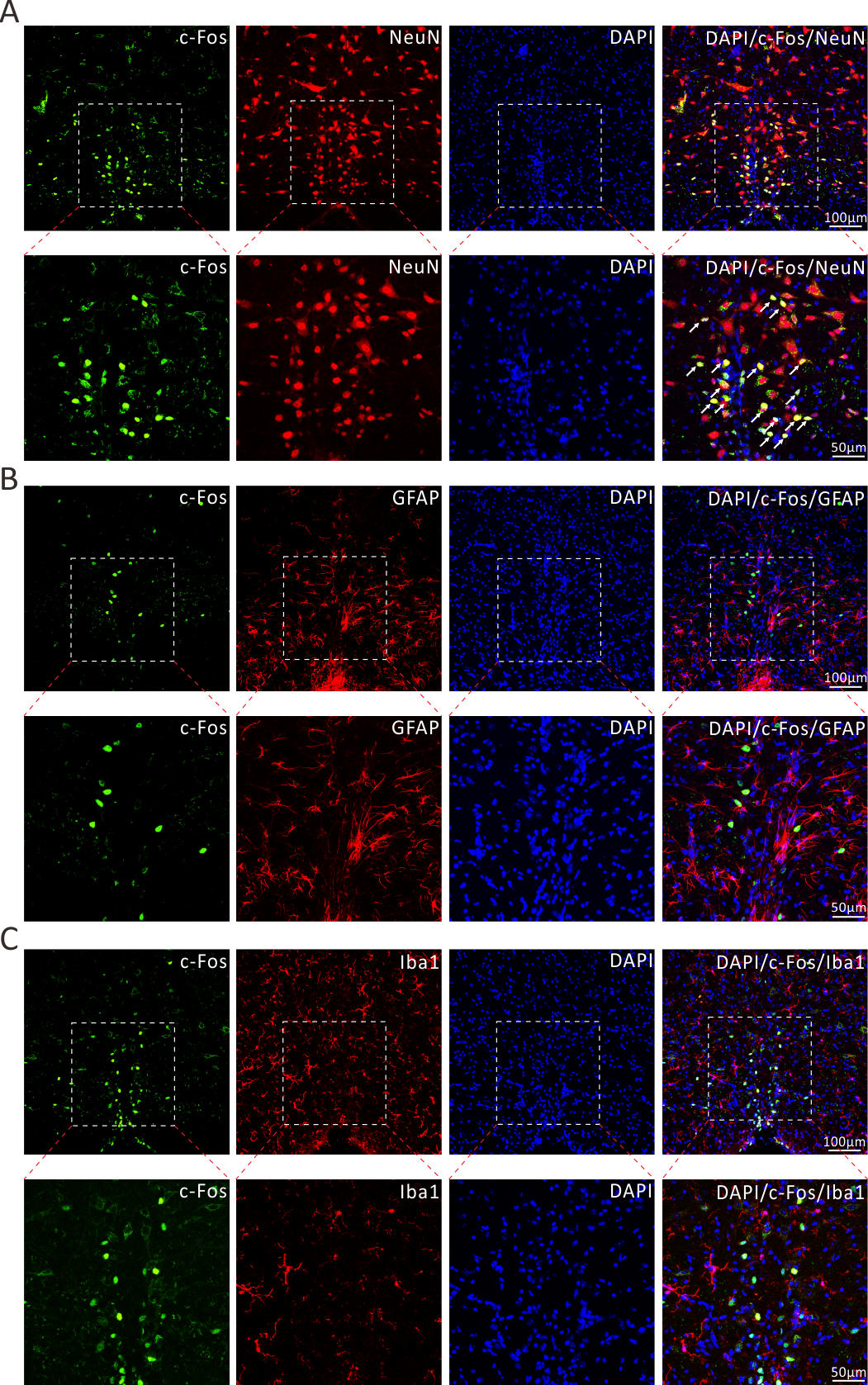

Supplement: Supplementary file 3 — Fig S3 [file CNS-27-1313-s006.tif]

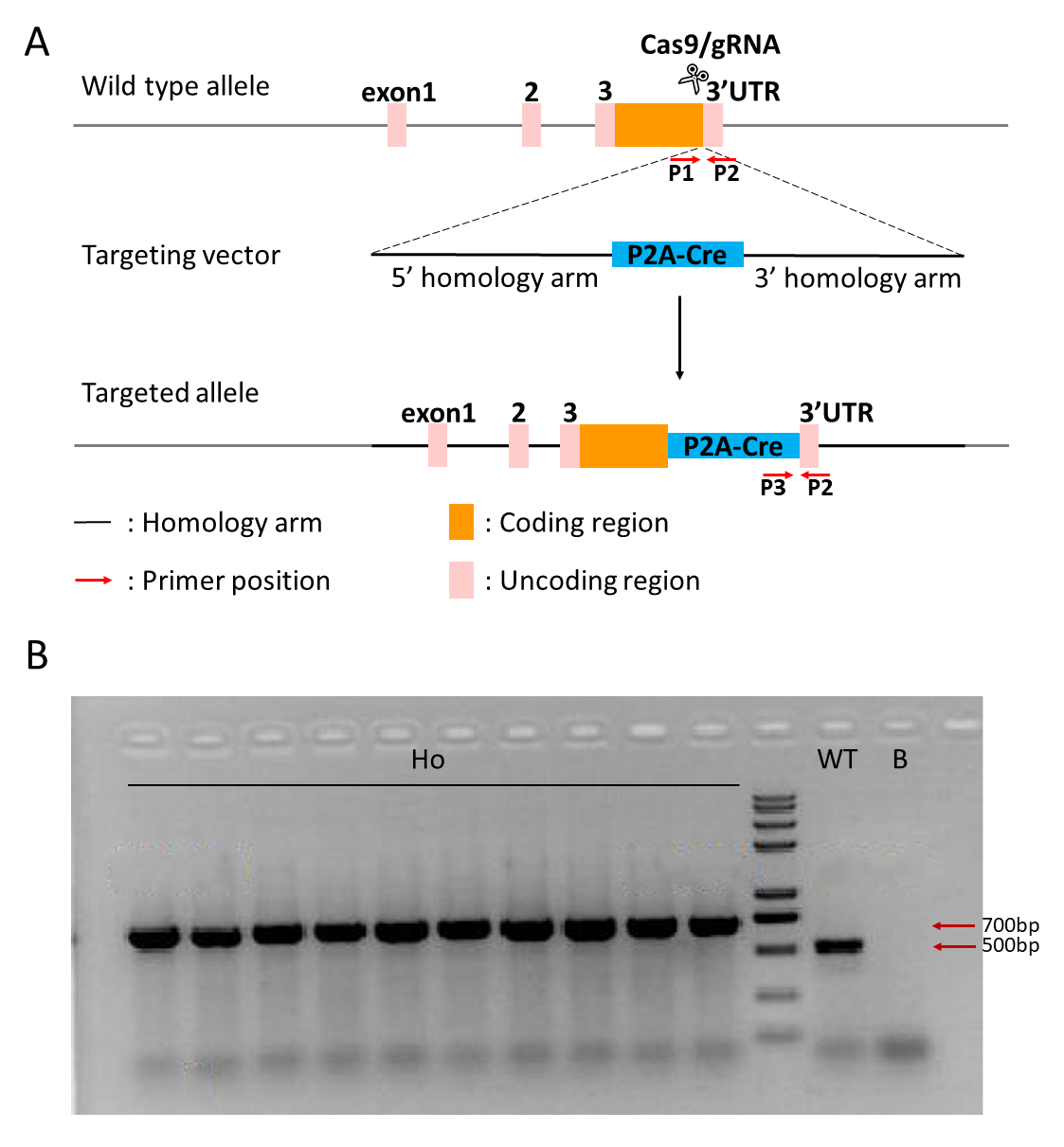

Supplement: Supplementary file 4 — Fig S4 [file CNS-27-1313-s002.tif]

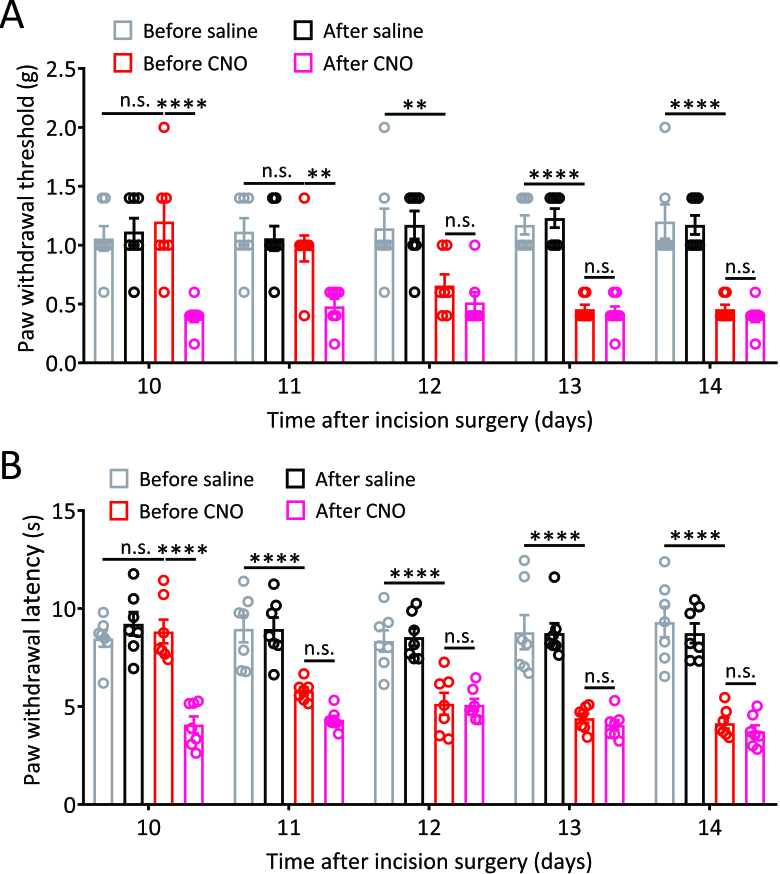

Supplement: Supplementary file 5 — Fig S5 [file CNS-27-1313-s009.tif]

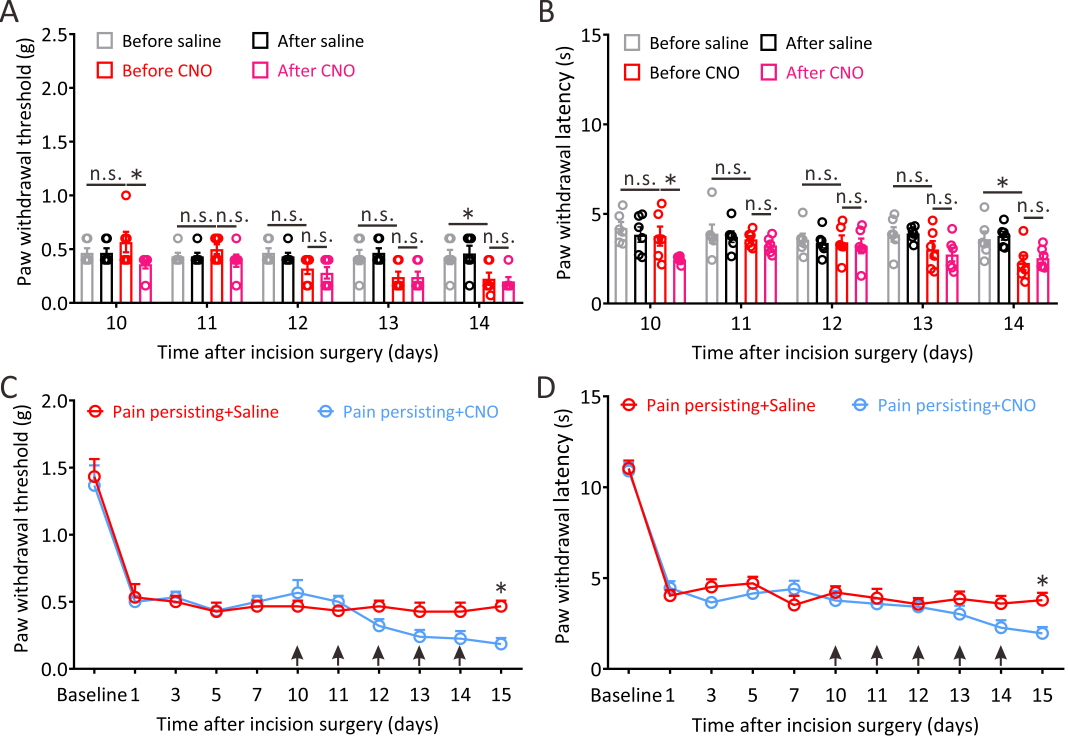

Supplement: Supplementary file 6 — Fig S6 [file CNS-27-1313-s003.tif]

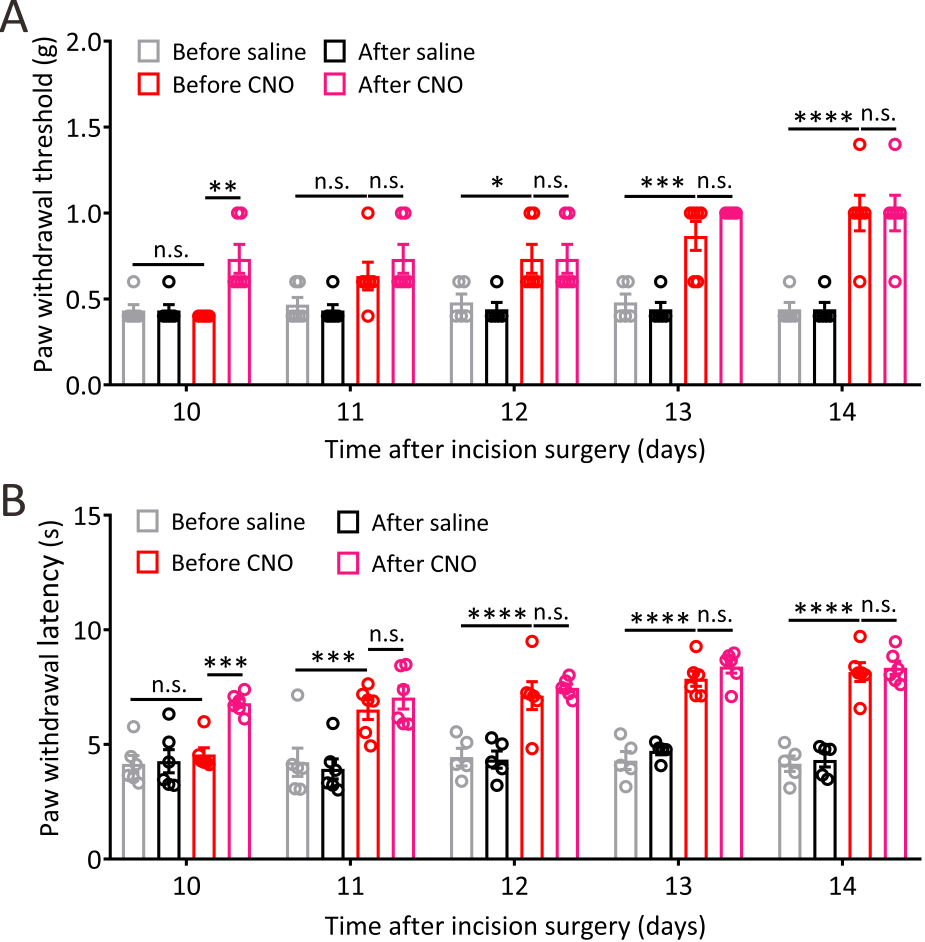

Supplement: Supplementary file 7 — Fig S7 [file CNS-27-1313-s005.tif]

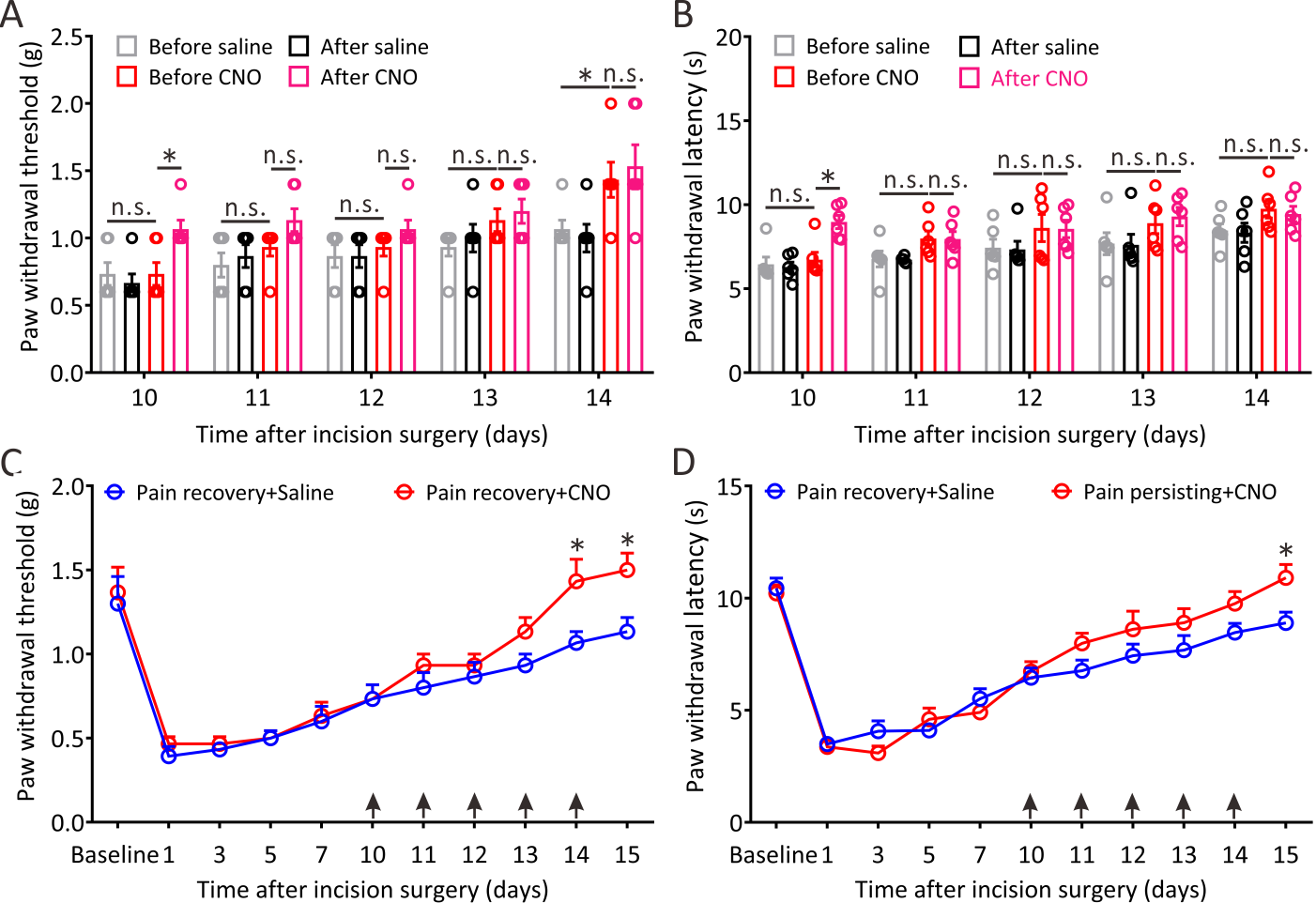

Supplement: Supplementary file 8 — Fig S8 [file CNS-27-1313-s001.tif]

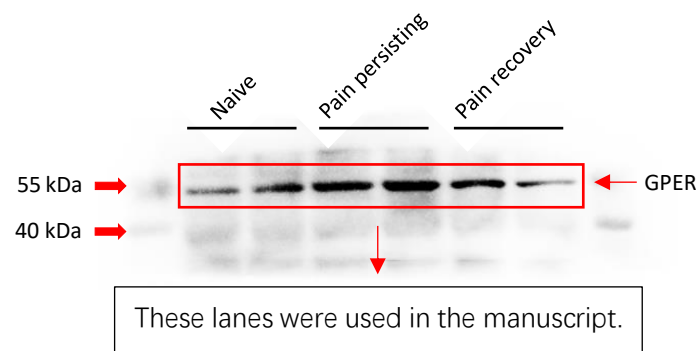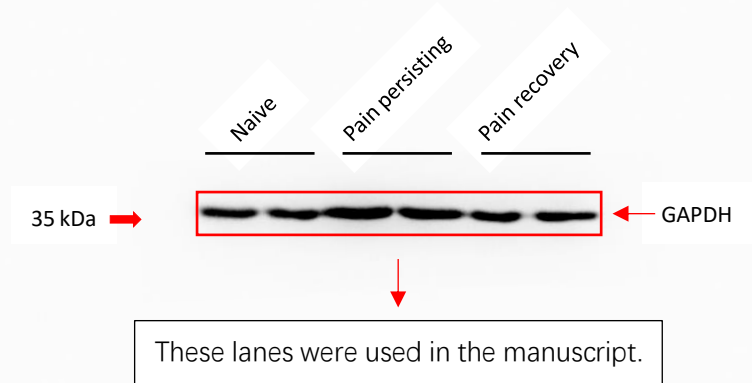

Full unedited blot for Figure 2C

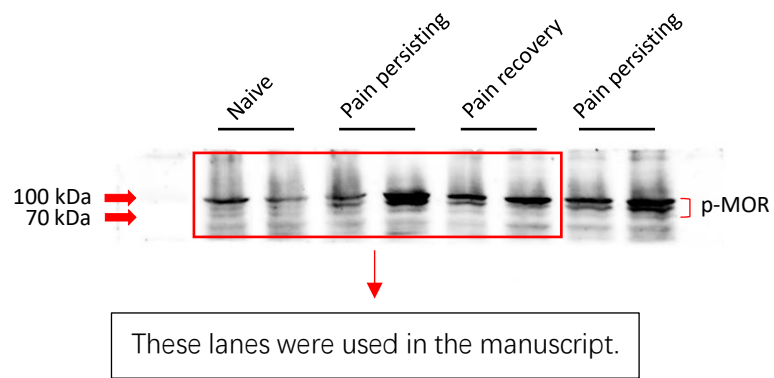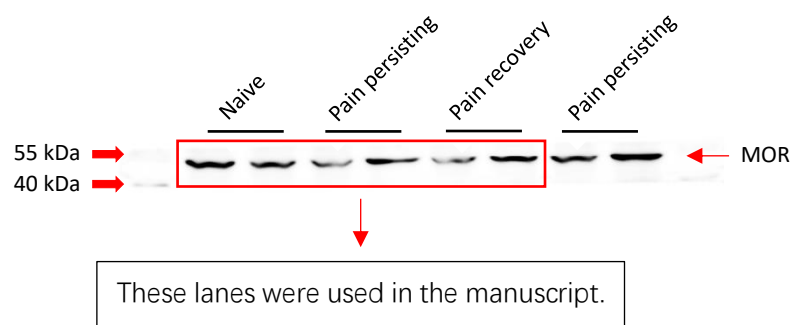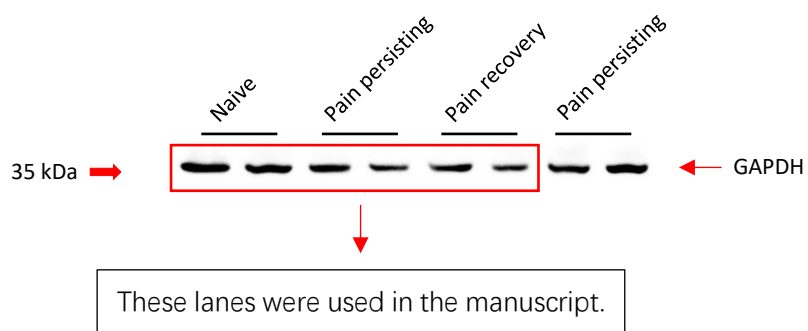

Full unedited blot for Figure 4A

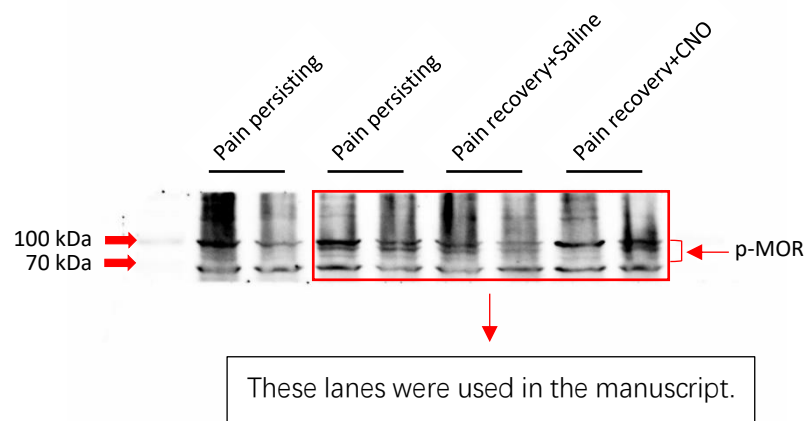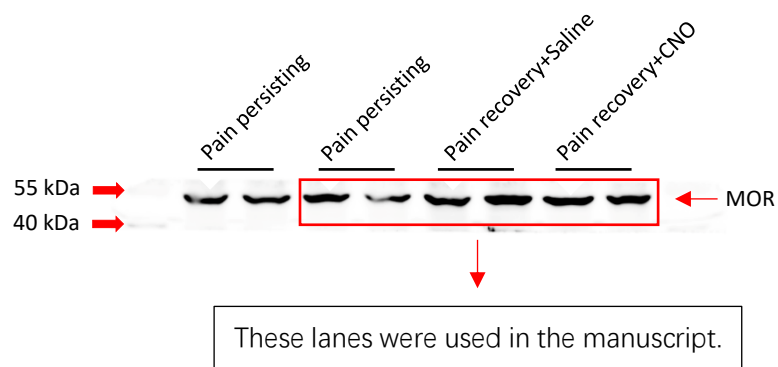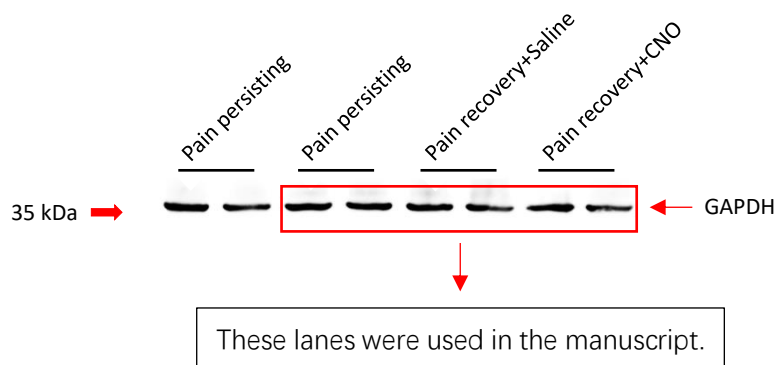

Full unedited blot for Figure 5H

Supplement: Supplementary file 9 — Supplementary Material [file CNS-27-1313-s007.pdf]
